# The influence of the stiffness of GelMA substrate on the outgrowth of PC12 cells

**DOI:** 10.1042/BSR20181748

**Published:** 2019-01-18

**Authors:** Yibing Wu, Yang Xiang, Jiehua Fang, Xiaokeng Li, Zunwen Lin, Guangli Dai, Jun Yin, Peng Wei, Deming Zhang

**Affiliations:** 1 The State Key Laboratory of Fluid Power and Mechatronic Systems, School of Mechanical Engineering, Zhejiang University, Hangzhou 310028, China; 2 Key Laboratory of 3D Printing Process and Equipment of Zhejiang Province, School of Mechanical Engineering, Zhejiang University, Hangzhou 310028, China; 3 Department of Hand and Foot Microsurgery, Ningbo First Hospital, Ningbo 315010, China; 4 Orthopedic department, The First Affiliated Hospital of Nanchang University, Nanchang 330006, China; 5 Department of Medical Engineering, Ningbo First Hospital, Ningbo 315010, China

**Keywords:** cell viability, cell spreading, gelatin methacryloyl, PC12 cells, substrate Young’s modulus

## Abstract

Recent studies have shown the importance of cell–substrate interaction on neurone outgrowth, where the Young’s modulus of the matrix plays a crucial role on the neurite length, migration, proliferation, and morphology of neurones. In the present study, PC12 cells were selected as the representative neurone to be cultured on hydrogel substrates with different stiffness to explore the effect of substrate stiffness on the neurone outgrowth. By adjusting the concentration of gelatin methacryloyl (GelMA), the hydrogel substrates with the variation of stiffnesses (indicated by Young’s modulus) from approximately 3–180 KPa were prepared. It is found that the stiffness of GelMA substrates influences neuronal outgrowth, including cell viability, adhesion, spreading, and average neurite length. Our results show a critical range of substrate’s Young’s modulus that support PC12 outgrowth, and modulate the cell characteristics and morphology. The present study provides an insight into the relationship between the stiffness of GelMA hydrogel substrates and PC12 cell outgrowth, and helps the design and optimization of tissue engineering scaffolds for nerve regeneration.

## Introduction

The treatment of peripheral nerve injury is a global clinical problem, which causes enormous economic burden to the society [1]. Crush injuries, penetration, ischemia, traction as well as radiation, vibration and electric shock are common causes of peripheral nerve injuries [2]. Severe peripheral nerve injury, which leads to the loss of sensory and motor functions of the innervated limbs and peripheral neuropathic pain, has a devastating impact on patients’ life quality. The development of tissue-engineered scaffolds has become a popular current avenue to repair peripheral nerve injuries [3,4]. In order to improve the efficiency of these treatments, it is essential to have a better understanding of how nerve cells interact with scaffolds and how these interactions affect cellular behavior. Since the stiffness of scaffold material varies significantly, the scaffold stiffness may play an important role in nerve regeneration.

Recent work has shown the importance of the substrate stiffness on the neurite length, migration, proliferation, and morphology of various nerve cell types [5]. For example, neural stem/progenitor cell (NSPC) cultured on photocrosslinkable methacrylamide chitosan hydrogels, where the concentrations of photoinitiator and solvent were varied to control the Young’s modulus, indicating that the variation of substrate Young’s modulus can alter cell differentiation and proliferation [6]. Primary rat cortical neurones (RCN) were cultured on soft and stiff substrates with Young’s modulus of 5–500 KPa, respectively, to investigate the role of the matrix rigidity on the formation and activity of cortical neuronal networks, and the results showed that migration of cortical neurones is enhanced on soft substrates, leading to a faster formation of neuronal networks [7]. Embryonic and adult neuronal progenitor cells were cultured on bifunctionalized IKVAV/PL substrates with different values of Young’s modulus, and the results showed that cell viability, average axon length, the number of dendritic filopodia and secondary branches were significantly improved on 2 KPa IKVAV/PL gels compared with the other Young’s modulus regions [8]. These studies have demonstrated that the substrate stiffness or the intrinsic elasticity of the matrix is emerging as a critical physical factor to influence nerve response. Therefore, a strong understanding of the relation between the substrates stiffness and nerve regeneration would be very useful in designing scaffolds with optimal mechanical properties.

The substrates are prepared to mimic the function of extracellular matrix (ECM) to provide a temporary place for cell growth, therefore, the material properties of substrate should be rigorously selected [9]. There are quite different biomaterials that have been developed for engineering cell microenvironment *in vitro* [10–13]. Gelatin methacryloyl (GelMA) hydrogels have been widely used for various biomedical applications due to their suitable biological properties and tunable physical characteristics [14–17]. The Young’s modulus of GelMA hydrogels can be modified by varying the hydrogel concentration, which are tuned from a few to hundreds of KPa [18]. Although, GelMA hydrogels have been utilized as substrate materials for cell culture; there are very few studies working on the relationship between the stiffness of GelMA hydrogel substrates and the growth characteristics and morphology of nerve cells.

In this work, substrates with different values of Young’s modulus were fabricated by varying the concentration of GelMA. Uniaxial tensile tests were carried to measure the value of Young’s modulus of the GelMA hydrogels. PC12 cells were cultured on the GelMA substrates with different values of Young’s modulus, and the cellular viability, adhesion and spreading, and the average neurite length were measured. Our results show a critical range of the stiffness of GelMA substrates that modulate and support the growth of PC12 cells and provide an insight into the relationship between the stiffness of GelMA substrates and the growth characteristics and morphology of PC12 cells.

## Materials and methods

### Synthesis of GelMA

GelMA was synthesized as described in the previous literature [15,19]. Briefly, GelMA was synthesized by the direct reaction of gelatin with methacrylic anhydride (MA). MA was added at a rate of 0.5 ml/min with vigorously stirring to form a 10% (w/v) solution of gelatin in PBS (pH = 7.5). After the reaction at 50°C for 3 h, the mixture solution was diluted and dialyzed for 7 days against distilled water at 40°C to remove methacrylic acid and anhydride, and filtered through a 0.22-μm membrane. Finally, the dialyzed solution was freeze-dried leading to a white solid and then stored at −80°C. The substitution degree of GelMA was controlled by analogous synthesis by changing the amount of MA [20], and the substitution degree of GelMA used in this work was approximately 96%.

**Figure 1:**
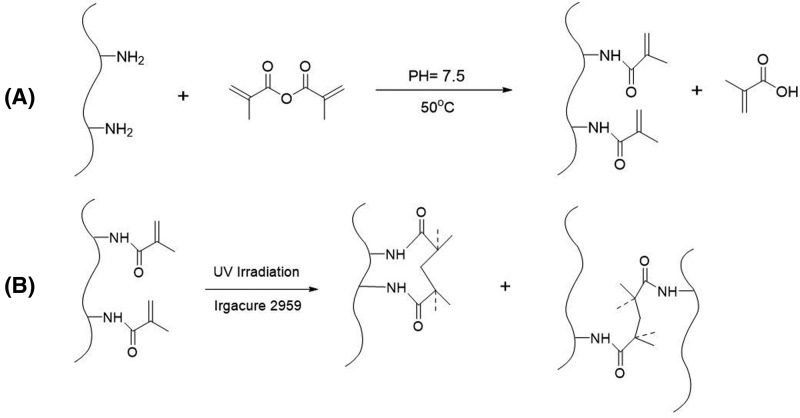
The schematic of the synthesis of GelMA process (**A**) Synthesis process of GelMA, and (**B**) photocrosslinking of GelMA hydrogel.

To form GelMA hydrogels with different concentrations (5, 10, 20, and 30%), GelMA precursor solutions were prepared by dissolving freeze-dried GelMA in deionized water at 40°C and maintaining for 2 h to form homogeneous solutions. The photoinitiator (Irgacure 2959) was then added to the precursor solutions at a concentration of 0.5% (w/v). Then, the solutions were exposed to a UV light with 1.5 mW/cm^2^ intensity and 365 nm wavelength for photocrosslinking of 30 s. The schematic of the synthesis process is as shown in Figure 1.

### Mechanical properties

**Figure 2:**
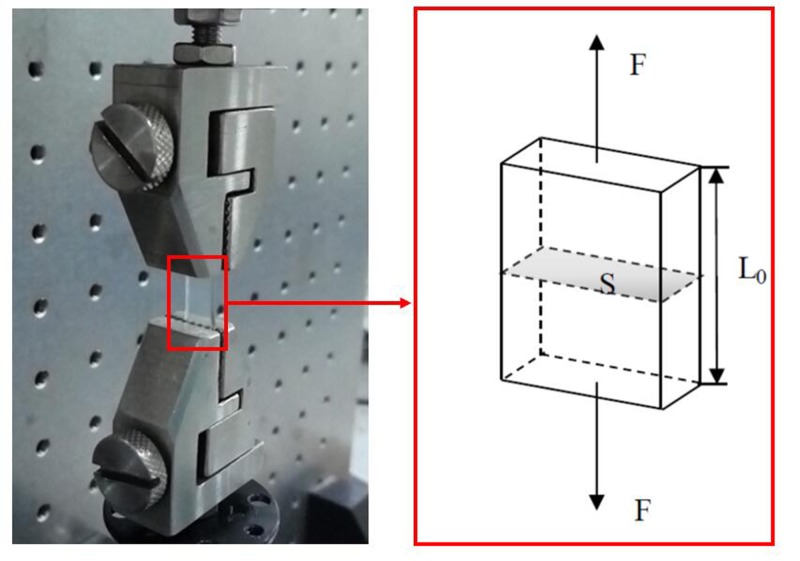
Experimental setup of the uniaxial tensile tests

The stiffness of GelMA hydrogels were characterized by the uniaxial tensile tests using a dynamic mechanical analysis instrument (ElectroForce, TA Instruments, U.S.A.) under room temperature. Rectangle specimens with the size of 20 × 5 mm^2^ and thickness of 3 mm were prepared. The samples of GelMA hydrogels was clamped with two steel clamps at a distance of 5 mm, and the uniaxial stretch rate was 1 mm/min (Figure 2). The Young’s modulus was determined by calculating the slope at the inititiation of the stress–strain curve. At least three specimens were used for each testing condition.

### Cell culture

The PC12 cell lines were derived from rat pheochromocytoma, a tumor arising from chromaffin cells of the adrenal medulla [21]. PC12 cells have been widely used *in vitro* neuronal model for the investigation of neuronal proliferation, differentiation, and cell viability [22–24]. PC12 cells were obtained from Cell Bank of the Chinese Academy of Science (Shanghai, China) and cultured in Dulbecco’s modified Eagle’s medium (DMEM, HyClone, supplemented with 10% FBS (Gibco, U.S.A.)) in an incubator (Thermo Scientific, U.S.A.) at 37°C with 5% CO_2_. PC12 cells with a concentration of 8 × 10^4^ cells/ml were harvested from cell culture dishes and were loaded on the GelMA substrates with different concentrations (5, 10, 20, and 30%).

After incubation for 24, 72, and 120 h, the cell-seeded hydrogel disks were rinsed with PBS and incubated with 1 µg/ml Calcein-AM and 5 µg/ml propidium iodide (PI) for 30 min to remove unattached cells. Then, the samples with attached cells were stained with 1 μg/ml calceinacetoxymethyl ester and 2 μg/ml PI for 30 min. The hydrogels were then imaged using a fluorescence microscope (Nikon Tis, Japan). The outgrowth characteristics and morphology of PC12 cells were analyzed by ImageJ software.

**Figure 3:**
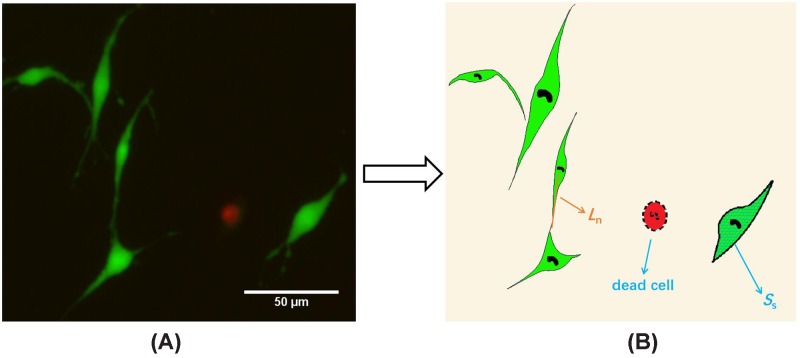
Definitaion the outgrowth characteristics and morphology of PC12 cells (**A**) Live-dead staining of live cells (green) and dead cells (red) in PC12 after 3 days of culture on 10% GelMA substrate. (**B**) The distance from the center of the cell to the end of the neurite (the orange line) represents length of neurite (*L_n_*). The area of cell contour (the blue grid) represents spreading area (*S_s_*).

Six cell-culture plates were performed for each group of GelMA substrates with different concentrations (5, 10, 20, and 30%). As illustrated in Figure 3, the distance from the center of the cell to the end of neurite (the orange line in Figure 3B) represents the length of neurite (*L_n_*), the area of cell contour (the blue grid in Figure 3B) represents the spreading area (*S_s_*) of the cell, and the number of live cells (*N_l_*) and dead cells (*N_d_*) can be obtained. The cell adhesion rate was defined as the number of live PC12 cells on substrate per cm^2^ [25,26]. Then the average length of neurite, the cell spreading area, the cell viability and the cell adhesion rate of each substrate were calculated. It should be noticed that only when *L_n_* > 5 µm and neurites do not contact other cells or neurites, those neurites were included in the neurite length analysis. Cell survival rate (*R_s_*) was defined as:

(1) $$R_{s}=\frac{N_{l}}{N_{l}+N_{d}}\times 100\%$$

### Statistical analysis

Unless otherwise stated, all characterizations were performed using data analysis software SPSS 18.0 (IBM, U.S.A.), and all data were presented in the form of mean ± S.D. Statistical significance (*P<0.05, **P<0.01) were determined using a Bonferroni’s multiple comparison one-way ANOVA. At least three independent experiments were performed for each condition in the present study.

## Results

### Hydrogel properties

**Figure 4:**
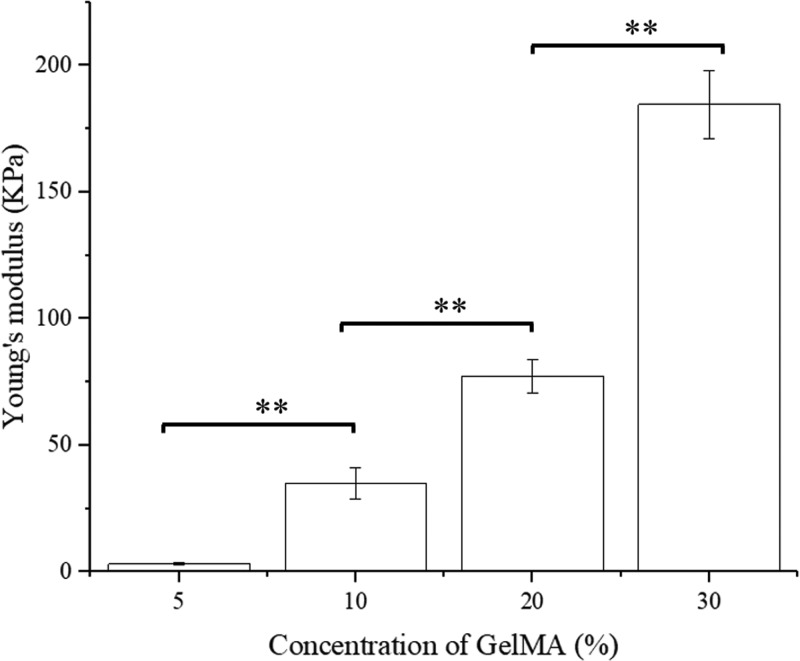
The Young’s modulus of GelMA hydrogels with different concentrations Bars represent average ± S.D. **P<0.01.

Figure 4 shows the influence of the GelMA concentration on the Young’s modulus of GelMA substrates. The 5% GelMA hydrogels were very soft with the Young’s modulus of only 3.08 KPa, and these hydrogels were even difficult to make a regular band bulk for the uniaxial tensile tests. The 10% of GelMA hydrogels were much stiffer with the Young’s modulus of 34.9 KPa which is ten-times larger than 5% GelMA hydrogels. Indicating that the stiffness of the hydrogels was evidently reinforced by the GelMA concentration. It needs to be noticed that compared with other three hydrogels, 30% of GelMA hydrogels had the largest Young’s modulus with the value of 184.52 KPa. As shown in Figure 4, the Young’s modulus significantly increased when the GelMA concentration increased from 5 to 30%.

### Cell culture

**Figure 5:**
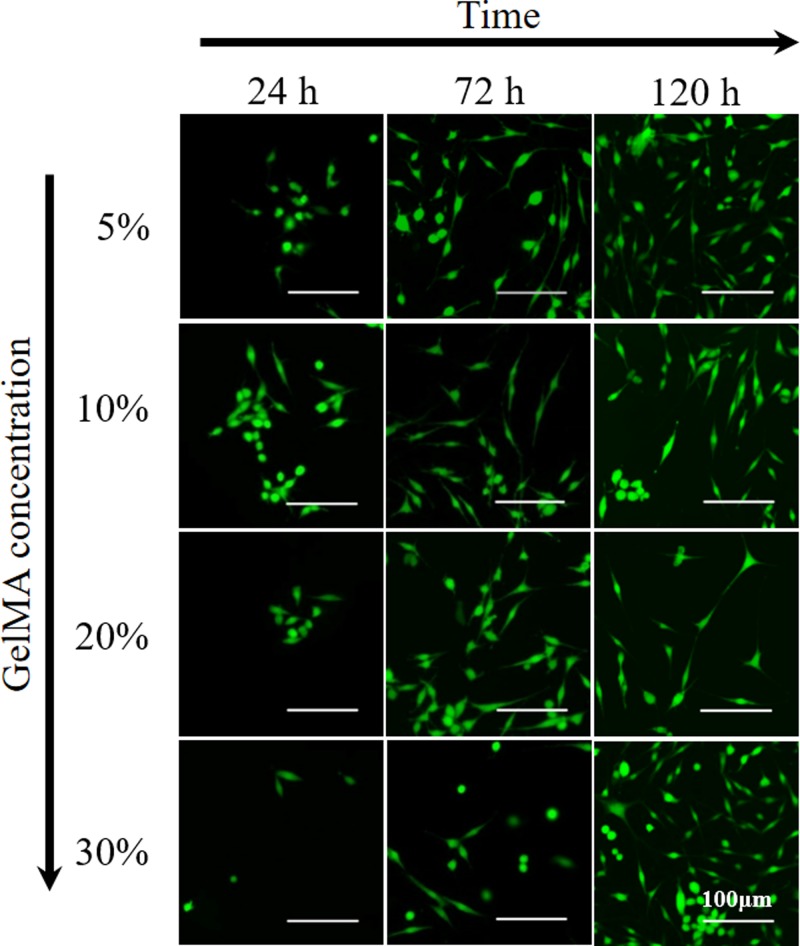
The presentative fluorography of PC12 cells adhesion on the surfaces of GelMA hydrogel substrates with different concentrations

PC12 cells were plated on the four groups of GelMA substrates with different moduli and found to adhere and spread on GelMA hydrogels. Figure 5 shows the representative outgrowth of PC12 cells adhesion on the surface of 5, 10, 20, and 30% concentrations of GelMA substrates, respectively. Within the first 24 h, PC12 cells showed similar morphology with very short neurites on all GelMA substrates. PC12 cells grew freely and the outgrowth of neurites were homogeneous in all directions after 72 and 120 h, exhibited vigorous spreading. The cell density also significantly increased from 24 to 120 h.

### Cell viability and adhesion

**Figure 6:**
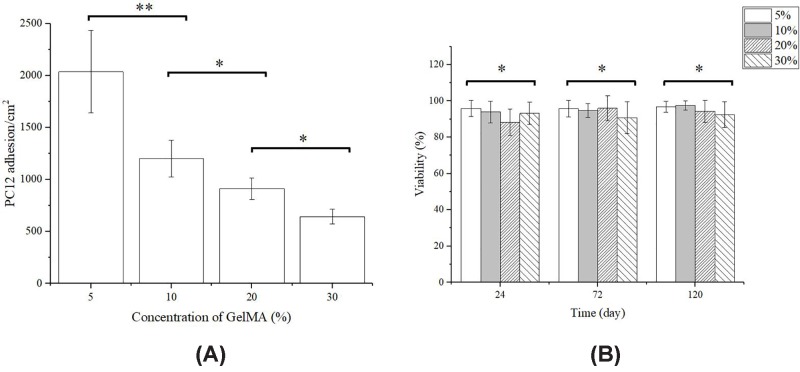
PC12 cells adhesion and viability (**A**) PC12 cells adhesion on GelMA substrates with different concentrations after 24 h of inoculation. (**B**) Cell viability after 24, 75, and 120 h of inoculation. Bars represent average ± S.D. (*P<0.05, **P<0.01).

To visualize the PC12 cells which were surviving and attached to the surface of the GelMA substrate, the samples after incubating for 24  h were characterized by cell adhesion rate and cell viability assay. Figure 6A shows the influence of the GelMA concentration on the PC12 cells adhesion. PC12 cells on the 5% GelMA substrates show the biggest adhesion rate of 2035 cells/cm^2^, which is twice the cell density on 20% GelMA substrates (912 cells/cm^2^) and triple the cell density on 30% GelMA substrates (643 cells/cm^2^). Therefore, PC12 cell adhesion rate decreases with the stiffness of GelMA substrates (Figure 6A).

As Figure 6B shows, cell viability of all groups at 24–120 h was approximately 95%, showing no significant difference (24 h: *P*=0.129; 72 h: *P*=0.887; 120 h: *P*=0.851, one-way ANOVA tests). It is obvious that GelMA hydrogel substrates are non-toxic to PC12 neuronal cells and suitable for PC12 culturing.

### Cell spreading area and neurite length

**Figure 7:**
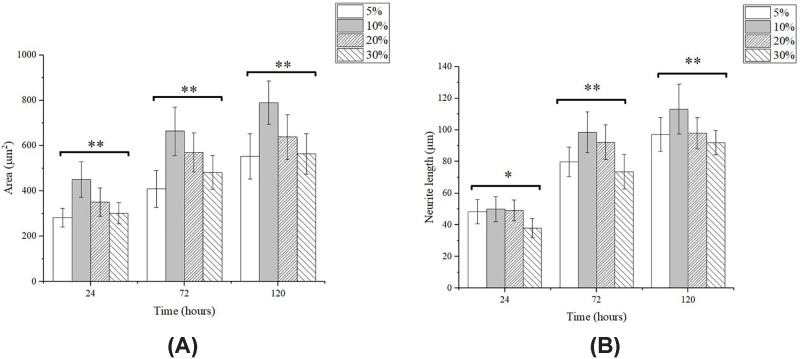
PC12 cells spreading area and neurite length Cell spreading area (**A**) and neurite length (**B**) of PC12 cells after 24, 75, and 120 h of inoculation. Points represent average ± S.D. (*P<0.05, **P<0.01).

Figure 7A shows the influence of the GelMA concentration of substrates on the spreading area of PC12 cells. It is shown that the cell spreading area in all groups increased significantly with time. The cell spreading area of PC12 cells was the smallest on 5% GelMA substrates, and was significantly improved on 10% GelMA substrates, when the Young’s modulus increased from 3.08 to 34.90 KPa. The neurite length of PC12 cells on GelMA substrates is shown in Figure 7B. The neurite length increased significantly with time but obvious differences were found at the different concentrations of GelMA hydrogel substrates. After incubating for 24 h, PC12 cell shows the longest neurite length on 10% GelMA substrates with 34.9 KPa, which was 49.9 μm, and not obviously different from neurite length on 5% (48.3 μm) and 20% (49.2 μm) GelMA substrates. Interestingly, neurite length on 10% GelMA substrates with 34.9 KPa was much longer than other three groups in 72 and 120 h. It can be found that as the Young’s modulus of the substrate increases, the cell spreading area and the neurite length first increase and then decrease.

## Discussion

Tissue engineering has been widely used to bridge peripheral nerve gaps, where the desired outcome is nerve tissue regeneration and functional recovery [1]. The successful nerve regeneration relies on the extensive growth of axonal processes [27]. The goal of this work is to explore the effect of the substrate stiffness on the neurone outgrowth. As previous work has shown that the mechanical properties of photocrosslinked GelMA hydrogels could be effectively regulated by increase in GelMA concentration [28]; in this work, the Young’s modulus of GelMA hydrogels increased from 3.08 to 184.52 KPa, when GelMA concentration increased from 5 to 30%. Based on PC12 cell culture on GelMA substrates, we found cell attachment amount was greatest on the softest GelMA hydrogels (5% GelMA hydrogel). The same tendency was also reported by the previous study by Evans et al. [29] which found that when TG2α E14 embryonic stem cells cultured on polydimethylsiloxane (PDMS) substrates with the Young’s modulus ranging from 0.041 to 2.7MPa, cell attachment was greatest on the softest PDMS (0.041 MPa) after 24 h.

But the influence of substrate stiffness on the neurite length was found to be more complex that in this work, the neurite length of PC12 cells was found to first increase and then decrease with GelMA concentration of substrates, and the longest neurite length of PC12 cells appears on 10% GelMA substrates with Young’s modulus of 34.9 KPa. Thus, the longest neurite length of PC12 cells appears on intermediate substrate stiffness. Similar tendency was also reported by previous studies. Koch et al. [30] cultured dorsal root ganglion neurones (DRG) on polyacrylamide substrates with the Young’s modulus from 150 to 5000 Pa, DRG displayed maximal neurite length on substrate with the Young’s modulus of 1000 Pa. The elongation of neurite length is effectively regulated by growth cones [31]. The neuronal growth cone controls the direction and rate of axonal outgrowth by navigating the surrounding environment searching for molecular, mechanical, and topographical cues [32]. Growth cones can sense substrate stiffness through actin cytoskeleton, and interact with the myosin polymerized at its terminal to form retrograde fibrillar actin (F-Actin) flow. The strength of the coupling between F-actin flow and substrate determines the power of traction force, and this kind of coupling is affected by the stiffness of the environment. Franze et al. [33] described these couplings as focal adhesions (FA) and their formation and disintegration may be related to certain stretch-activated ion channels, and the substrate stiffness can affect FA by adjusting these ion channels, which ultimately affects protrusion elongation.

The spreading area of PC12 cells also showed the similar trend as neurite length on GelMA substrates, which achieved the maximal spreading area on the 10% GelMA substrates. Rosso et al. [34] also investigated the effect of substrate stiffness on growth cone morphology of DRG, and the growth cone areas significantly increased from 75.8 ± 4 μm^2^ on 1 KPa substrates to 189.5 ± 20 μm^2^ on 20 KPa substrates, and no significant differences were observed amongst 1 KPa substrates, 10 KPa substrates, and glass substrates.

Here, we demonstrate that cell viability, spreading area, and neurite length of PC12 cells strongly depend on substrate stiffness. Moreover, the cell spreading area and the neurite length of PC12 cells were significantly improved on 34.90 KPa GelMA substrates compared with the other stiffness regions. It can be concluded that 10% GelMA hydrogel is the optimal for nerve regeneration of PC12 cell. Since GelMA has been used as artificial nerve conduits for peripheral nerve regeneration [35], this work would be helpful for further improving the repair efficiency of nerve regeneration.

## Conclusion

In the present study, we employed photocrosslinkable GelMA hydrogels to fabricate substrates for PC12 cell culture. The concentration of GelMA was demonstrated to be an effective approach to tailor the substrate stiffness. The Young’s modulus of GelMA substrates increased from 3.08 to 184.52 KPa with the GelMA concentration from 5 to 30%. GelMA hydrogels were confirmed to have an excellent biocompatibility for PC12 cells. As the stiffness of the substrate increased, PC12 cell adhesion rate monotonous decreased, however the cell spreading area and the neurite length first increased and then decreased. The 10% GelMA hydrogel is optimal for nerve regeneration of PC12 cell. Our results show a critical range of the GelMA substrates stiffness that modulate and support the growth of PC12 cells and provide an insight into the relationship between the stiffness of GelMA substrates and the outgrowth characteristics and morphology of PC12 cells.

## Abbreviations

DRG: dorsal root ganglion neurone
FA: focal adhesion
F-Actin: fibrillar actin
GelMA: gelatin methacryloyl
MA: methacrylic anhydride
PDMS: polydimethylsiloxane
PI: propidium iodide
